# Delivering genes with human immunodeficiency virus-derived vehicles: still state-of-the-art after 25 years

**DOI:** 10.1186/s12929-022-00865-4

**Published:** 2022-10-09

**Authors:** Jonas Holst Wolff, Jacob Giehm Mikkelsen

**Affiliations:** Department of Biomedicine, Aarhus University, Høegh-Guldbergs Gade 10, 8000 Aarhus C, Denmark

**Keywords:** HIV, Lentivirus, Lentiviral vectors, Integrase-defective lentiviral vectors, IDLV, Gene therapy, Gene editing, Genome engineering, CRISPR

## Abstract

Viruses are naturally endowed with the capacity to transfer genetic material between cells. Following early skepticism, engineered viruses have been used to transfer genetic information into thousands of patients, and genetic therapies are currently attracting large investments. Despite challenges and severe adverse effects along the way, optimized technologies and improved manufacturing processes are driving gene therapy toward clinical translation. Fueled by the outbreak of AIDS in the 1980s and the accompanying focus on human immunodeficiency virus (HIV), lentiviral vectors derived from HIV have grown to become one of the most successful and widely used vector technologies. In 2022, this vector technology has been around for more than 25 years. Here, we celebrate the anniversary by portraying the vector system and its intriguing properties. We dive into the technology itself and recapitulate the use of lentiviral vectors for ex vivo gene transfer to hematopoietic stem cells and for production of CAR T-cells. Furthermore, we describe the adaptation of lentiviral vectors for in vivo gene delivery and cover the important contribution of lentiviral vectors to basic molecular research including their role as carriers of CRISPR genome editing technologies. Last, we dwell on the emerging capacity of lentiviral particles to package and transfer foreign proteins.

## Introduction

Acquired immunodeficiency syndrome (AIDS) was first diagnosed in 1981. The first reports describing five men from the Los Angeles area who suffered from serious infections caused by severe immunosuppression [1] were quickly followed by additional findings showing Kaposi sarcoma and opportunistic infections in larger groups of men [2]. The hitherto unrecognized deadly illness was described then as a disease with reduced cellular immunity leading to infections and malignant neoplasms. In 1983, researchers at the Pasteur Institute in Paris cultured T-cells from a patient with early AIDS symptoms and found reverse transcriptase (RT) activity in culture media indicative of an ongoing retrovirus infection [3]. What followed led to characterization of the retrovirus and documented the causal link between virus infection and development of AIDS [4]. The virus was soon after, in 1986, named human immunodeficiency virus type 1 (HIV-1) [5].

### A roller coaster ride with gammaretroviral vectors

The fast track to discovery of HIV-1 as the causing agent of AIDS was built on early pioneering studies of genetically simpler retroviruses, which are now classified as alpha- and gamma-retroviruses. Most importantly, in back-to-back publications published 50 years ago, Howard Temin and David Baltimore rewrote the central dogma by documenting that virions derived from two tumor-causing RNA viruses, Rous sarcoma virus (RSV) and Rauscher murine leukemia virus (R-MLV), carried a virally encoded enzyme capable of converting single-stranded RNA into DNA [6, 7]. Eventually, this allowed molecular details of DNA production from RNA templates to be uncovered [8], leading to an understanding of reverse transcription and genomic integration of double-stranded DNA as major hallmarks of retroviral replication.

The molecular characterization of simple retroviruses, like murine leukemia virus (MLV), did not only support the swift identification of HIV-1 as the causative agent of AIDS. It also paved the way for new concepts of gene therapy, which would start taking shape in the early 1980s and embark on a thrilling and often bumpy roller coaster ride through good and bad times for the next forty years. Along for the ride came vector technologies based on HIV in the late 1990’s, but it all started with work by the labs of Baltimore and Temin, who had now moved up the retroviral genome from the *pol* gene encoding the RT to a region near the 5′-end, where they stumbled upon the RNA motifs directing incorporation of viral RNA into virions.

The secondary RNA structures that direct RNA encapsidation into virions are collectively referred to as the packaging signal, Ψ. When Baltimore and colleagues removed a 351-nucleotide segment located downstream from the primer binding site in Moloney murine leukemia virus (Mo-MLV), they found that viral replication was severely suppressed [9]. Importantly, however, they also found that the defective virus was able to incorporate engineered heterologous RNA molecules—as long as the 351-nucleotide segment was present in these RNAs. With this rather simple setup they had not only identified the Mo-MLV Ψ sequence, but also established the fundamental concepts of retroviral vector technologies. Baltimore’s team went on to generate cell lines with stable expression of MLV proteins, which served as factories of virions incorporating Ψ-containing RNAs, and showed virus-based transfer of such RNAs to target cells leading to genomic integration of reverse-transcribed DNA. In parallel studies, Watanabe and Temin found a similar *cis*-acting defect in RSVs carrying a deletion at a similar position in the genome [10] and likewise demonstrated the production of replication-defective retrovirus vectors without the production of replication-competent helper virus [11].

Out of concurrent studies providing proof-of-principle for retroviral gene transfer to hematopoietic stem cells [12, 13] grew the vision of exploiting the capacity of retroviruses to transfer foreign genes as a new platform for genetic therapies of diseases amenable to hematopoietic stem cell transplantation. Although subsequent reports successfully demonstrated the concepts of hematopoietic stem cell therapy using retroviral gene transfer in animal models and supported a path toward clinical translation [14], a breakthrough in humans was challenged by technical issues related to culturing and expansion of stem cells, efficacy of gene transfer, and the capacity of retrovirally transduced cells to engraft. For treatment of severe combined immunodeficiency caused by adenosine deaminase deficiency (ADA-SCID), these issues were resolved one by one leading to evidence of immunological reconstitution in patients [15, 16]. Retrovirally transduced CD34^+^ hematopoietic stem cells derived from ADA-SCID patients were eventually developed to become a gene therapy product for autologous stem cell transplantation in patients lacking a matched hematopoietic stem cell donor. This medicine, designated Strimvelis, obtained marketing authorization by the European Medicines Agency in 2016 and was purchased by Orchard Therapeutics Ltd from GlaxoSmithKline in 2018.

Strimvelis is based on an early-generation MLV vector carrying an ADA cDNA expression cassette driven by the long terminal repeat (LTR) promoter. The capacity of this vector to insert into the genome is essential for its use to treat stem cells, but this inherent action of the vector is also the Achilles heel of the technology. Hence, a genomically inserted vector may disturb endogenous gene regulation and epigenetic marking or directly affect the reading frame of essential genes. We now know that MLVs have a tendency to insert near strong enhancers, active promoters and transcriptional start sites [13, 17–19], which increases the risk of insertional mutagenesis leading to activation of proto-oncogenes. This integration profile is driven by mechanisms that retroviruses have adapted to ensure active transcription of proviral DNA by inserting into transcriptionally active regions, and may therefore vary between cell types [20]. In the case of MLV, the bromodomain and extra-terminal (BET) family of proteins has been shown to interact with the virally encoded integrase [21, 22]. BET proteins help the preintegration complex tether to target DNA and are considered key determinants of the MLV insertion profile [23–25].

Despite the tendency of MLV vectors to insert into the regulatory regions of genes, severe toxicities related to insertional mutagenesis of MLV vectors used for treatment of ADA-SCID have not been reported in treated patients. However, very recently, in October 2020, Orchard Therapeutics Ltd announced that a patient treated with Strimvelis has been diagnosed with lymphoid T-cell leukemia and is undergoing treatment for the leukemia. It is possible that the diagnosis may be attributable to an event of vector insertion related to the gene therapy treatment, but more information is still needed. This announcement follows on reports indicating that insertion near proto-oncogenes and subtle mutagenic clonal variations are indeed observed in γ-retroviral gene therapy-treated ADA-SCID patients [26]. This unfortunate incidence follows in the wake of reports of vector-driven malignancies observed in related genetic therapies for other conditions. Among twenty patients treated for X-linked SCID (SCID-X1) using a similar approach of MLV-based transfer of an LTR-driven *IL2RG* expression cassette, five patients, who have clinically benefitted from the treatment, developed lymphoid T-cell leukemia. In four of these patients, malignancy was induced by insertional activation of the *LMO-2* protooncogene [27, 28]. Similarly, patients suffering from Wiskott-Aldrich syndrome treated with an LTR-driven MLV vector developed leukemia due to integration of the vector near the *LMO-2* gene [29].

To sum up, years of pioneering work and development had demonstrated the clinical potential of retroviral gene therapy, but also unveiled the serious side effects and downsides of the vector and its actions. Fortunately, new vector configurations were in the pipeline, and one of them was built on knowledge gained from the battle against AIDS.

### The birth and maturation of HIV-1-derived lentiviral vectors

Just like in MLV and RSV, sequences in the 5′ untranslated region of HIV-1 genomic region were found to be required for packaging of RNA into virions [30–32]. A series of early reports suggested that heterologous RNA transcripts containing the RNA motifs present in this region were packaged actively into virus particles [33–36] essentially providing the first examples of lentivirus-derived vectors. An early generation of helper virus-free vector transfer was achieved by expressing *gag-pol* and *env* genes from separate plasmids allowing gene transfer to CD4-positive cells, the native target cells of HIV-1 [37].

**Fig. 1:**
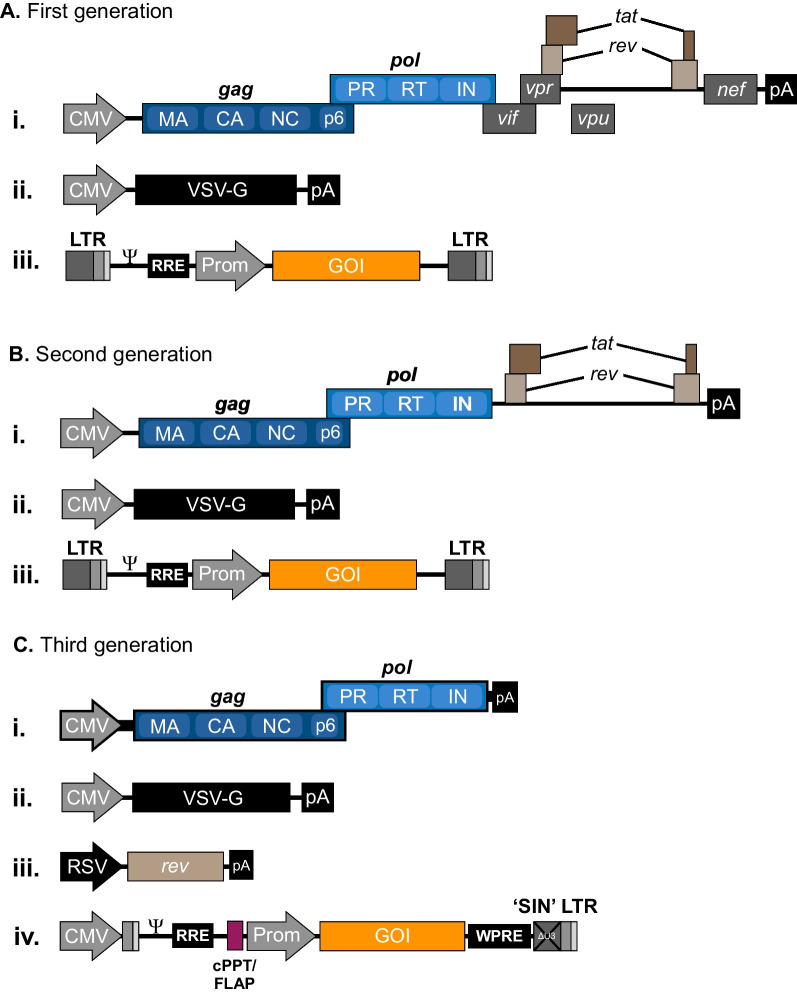
Schematic representation of the three generations of lentiviral vectors.** A** First generation vector systems include all HIV-1 genes, except *env*, in a single packaging plasmid. The *env* gene is replaced with VSV-G and provided in a separate plasmid. The vector plasmid contains an internal promotor-driven transgene cassette flanked by the HIV-1 LTRs. **B** In second-generation lentiviral vector systems, genes encoding accessory proteins Vif, Vpr, Vpu, and Nef are removed from the packaging plasmid. **C** In third-generation lentiviral vector system, the *rev* gene is placed on a separate plasmid, giving rise to a total of four separate plasmids required for production. Replacement of the U3 with a heterologous promoter (usually CMV or RSV) in the 5′ LTR allows the *tat* gene to be removed from the packaging plasmid, while a partial deletion of the U3 region from the 3′ LTR results in so-called ‘self-inactivating’ (SIN) lentiviral vectors. State-of-the art third-generation lentiviral transfer vectors usually also contains additional *cis*-acting elements such as cPPT/FLAP and WPRE for increased transduction efficiency and transgene expression, respectively. All vectors are shown schematically, and elements such as genes and promoters are not shown to scale

In 1996, Naldini and coworkers reported on a first-generation vector system (schematically shown in Fig. 1A) based on transient transfection of three plasmids, (i) a packaging construct with expression of Gag, GagPol, and several of the accessory proteins driven by a heterologous CMV promoter, but with blocked Env and Vpu reading frames, (ii) an Env-expressing plasmid encoding the vesicular stomatitis virus glycoprotein (VSV-G), and (iii) the lentiviral vector plasmid carrying an internal CMV-driven transgene expression cassette flanked by HIV-1 LTRs and all required *cis*-elements [38]. With this vector system, the authors demonstrated in vivo lentiviral gene transfer to neurons in rats and stable transduction of nondividing cells. As MLV-based systems lack the capacity to transduce nondividing cells, this finding unveiled an attractive new capacity to insert genes into the genomes of nonproliferating cells. An improved second-generation vector system utilizing a packaging construct devoid of the accessory genes *vif*, *vpr*, *vpu*, and *nef* (Fig. 1B) [39] was soon after replaced by a third-generation lentiviral vector system (Fig. 1C) [40], which would eventually become the standard platform for production of lentiviral vectors. To reduce the risk of producing replication-competent HIV-1 during viral vector production, the authors modified the packaging construct by removing the *tat*- and *rev*-encoding exons, allowing only Gag and GagPol to be produced from this plasmid. However, to produce Rev in trans, an additional plasmid encoding the Rev protein driven by a heterologous promoter was introduced. In addition, the U3-region containing the viral promoter in the 5’-LTR of the vector construct was replaced with a heterologous promoter, allowing the transcriptional site of HIV-1 to be preserved and vector RNA containing the *cis*-elements for reverse transcription and integration to be expressed independently of Tat. Work by Dull and coworkers resulted in two vector configurations, pRRL and pCCL, which would soon be distributed to labs worldwide and become standard lentiviral vectors. In pRRL, the 233-bp RSV enhancer/promoter was introduced instead of the viral promoter, whereas in pCCL, a 673-bp enhancer/promoter sequence from cytomegalovirus (CMV) replaced the original promoter [40].

**Fig. 2:**
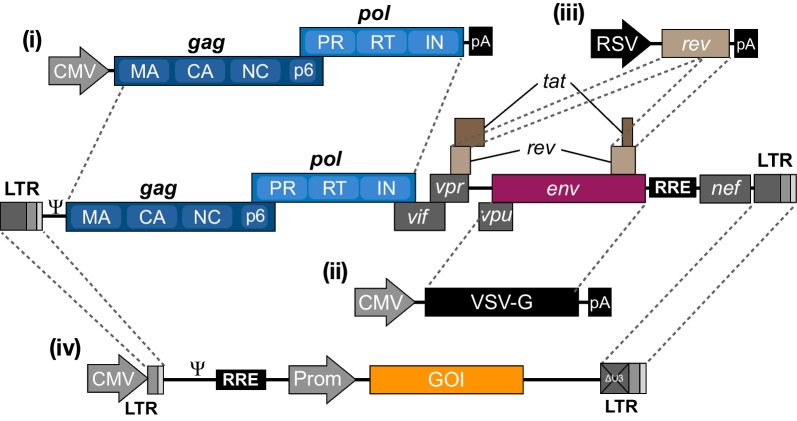
Derivation of current third-generation lentiviral vectors. The HIV-1 genome encodes three structural genes (*gag*, *pol* and *env*) as well as regulatory (*rev* and *tat*) and accessory (*vif*, *vpr*, *vpu* and *nef*) genes. The Gag precursor contains the viral core proteins, which are the matrix (MA), capsid (CA), nucleocapsid (NC) and p6 proteins, whereas the GagPol precursor also contains the protease (PR), reverse transcriptase (RT) and the integrase (IN) proteins. The entire HIV-1 genome is flanked by long terminal repeats (LTRs), responsible for viral transcription, reverse transcription and integration. For the production of current third-generation lentiviral vectors, the essential parts of the HIV-1 genome have been split into four separate plasmids; (i) the packaging plasmid encoding the GagPol polyprotein, (ii) the envelope plasmid encoding the viral glycoprotein (here VSV-G), (iii) the rev plasmid encoding Rev, and (iv) the transfer vector, carrying the transgene flanked by LTRs

**Fig. 3:**
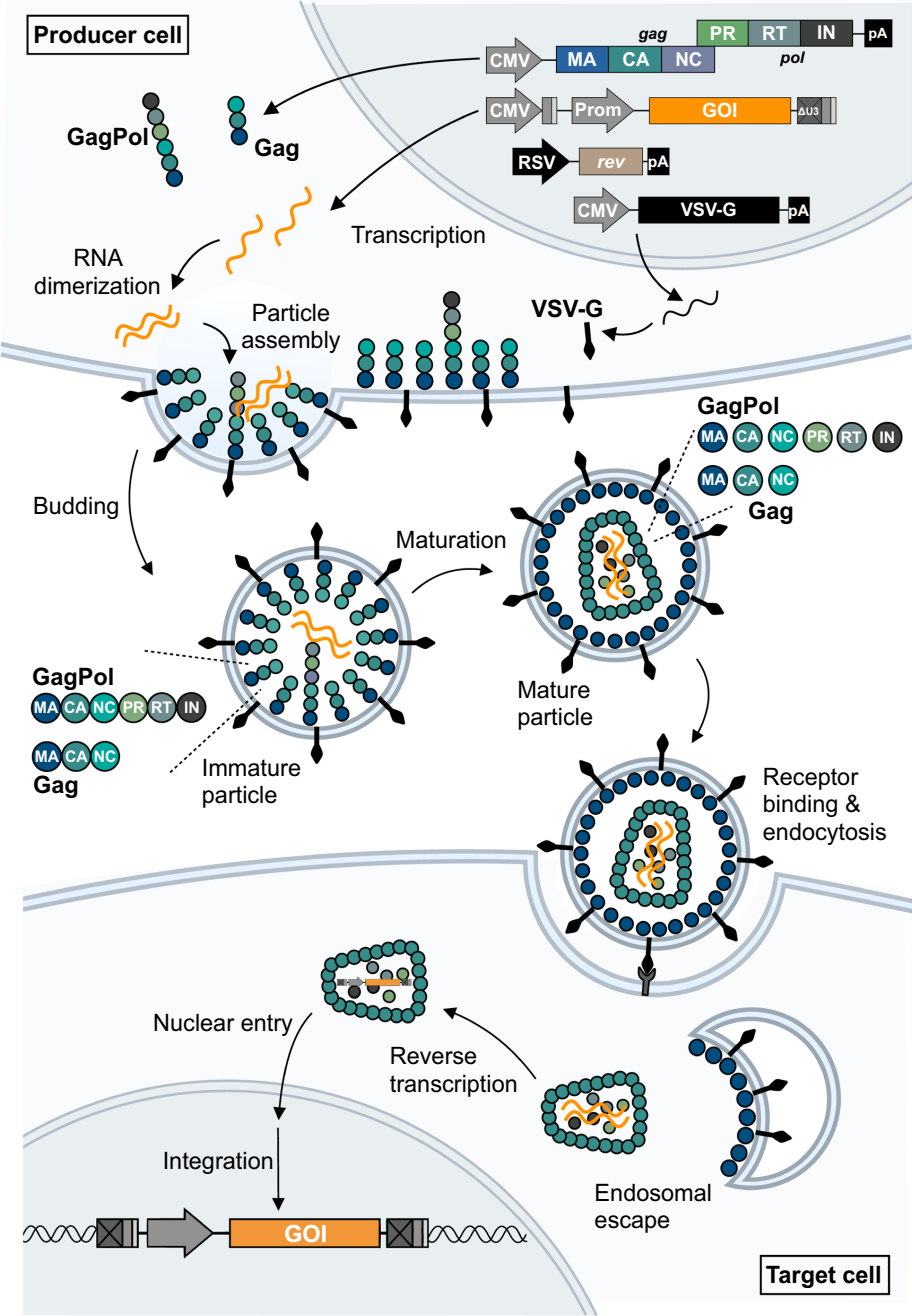
Gene transfer using lentiviral vectors. Third-generation lentiviral vectors are produced by transfecting producer cells with the four packaging plasmids, which will initiate transcription of Gag and GagPol polyprotein precursors, the envelope glycoprotein (e.g. VSV-G), Rev and the transfer vector carrying the (trans)gene of interest (GOI) to be inserted into the target cells. Nascent lentiviral particles are packaged together with an RNA dimer encoding the transgene flanked by viral *ci*s-elements required for RNA packaging and reverse transcription. Budding of lentiviral particles results in immature particles, which are then matured in a process involving cleavage of the Gag and GagPol polyproteins as well as formation of the viral core. Uptake into target cells is achieved through receptor-mediated endocytosis, following which the viral core is released into the cytoplasm. Reverse transcription of the transfer vector single-stranded RNA then occurs, resulting in double-stranded DNA, which is then transported into the nucleus and integrated into the genome of the target cell

By excluding six of nine genes present in the parental HIV-1 genome and expressing the remaining genes, *gag*, *pol*, and *rev* plus a heterologous *env* gene, from three separate plasmids (Fig. 2), the risk of generating replication-competent HIV-1 during third-generation vector production was minimized. Also, only vector RNA contained *cis*-elements required for RNA packaging, reverse transcription, and integration (Fig. 2), reducing the risk of recombination during reverse transcription. As of today, 25 years down the line, generation of replication-competent viruses has not been reported. Notably, however, although these vectors are considered safe, one should be aware of scenarios, for example in studies including HIV-1-infected primary cells, where recombination events involving vector RNAs and packageable HIV-1 transcripts could possibly lead to formation of replication-competent viruses with altered biological properties. In relation to this, attempts have been made to reduce the sequence homology between native HIV-1 and lentiviral vectors. Such attempts include replacing the RRE of lentiviral vectors with a heterologous RRE-like RNA element [41, 42], or splitting Gag/GagPol into separate packaging plasmids [43], although these approaches have not been widely adopted. The general setup for current third-generation lentiviral vector production and transduction is outlined in Fig. 3.

**Fig. 4:**
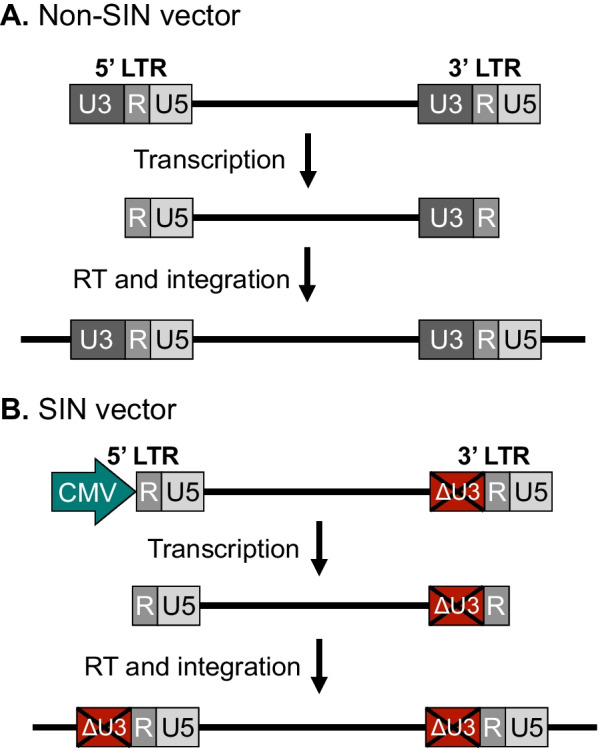
Self-inactivating (SIN) lentiviral vectors. **A** Non-SIN lentiviral vectors contain two wild-type HIV-1 LTRs, each comprised of a U3, R and U5 region. The U3 region contains promoter/enhancer regions from which transcription initiates at the junction between U3 and R in the 5′ LTR and terminates at the junction between R and U5 in the 3′ LTR. Viral transcripts thus lack the 5′ U3 and the 3′ U5, which are regained by duplication from each end upon reverse transcription. **B** In SIN lentiviral vectors, the 5′ U3 region is replaced by a heterologous promotor, such as CMV, whereas part of the 3′ U3 region has been deleted. When the 3′ U3 region of SIN vectors are duplicated, the deletion in the 3′ U3 (ΔU3) is transferred to the 5′ LTR, rendering the integrated lentiviral vector remote of viral promoter/enhancer regions

Early on, a few additional modifications to the third-generation vector design were introduced to increase safety and efficacy of lentiviral gene transfer. Firstly, to reduce the impact of integrated vectors, and in particular the 5′-LTR promoter, on the transcriptional activity of genes flanking the insert, a deletion of the viral promoter located in the U3 region of the 3’LTR was introduced in the plasmid DNA used to produce vector RNA [44]. The U3 region is copied during reverse transcription and is present therefore both in the 5′-LTR and 3′-LTR of proviral DNA (Fig. 4A). However, for vectors carrying the U3 deletion, referred to as ‘self-inactivating’ (or SIN) vectors, transcriptional activity of the proviral 5’-LTR is significantly reduced leading to reduced expression of full-length vector RNA in transduced cells (Fig. 4B) [45]. As none of the basic properties or efficacy of gene transfer were found to be compromised in SIN vectors [44], the SIN configuration quickly became a standard feature of lentiviral vectors. Secondly, Zufferey and colleagues addressed the tendency of some retrovirus-based vectors to support only relatively low levels of transgene expression. Zooming in on suboptimal nuclear export of transgene mRNA as a potential reason for reduced gene expression, they introduced the Woodchuck hepatitis virus posttranscriptional regulatory element (WPRE) in the 3’UTR of the transgene expression cassette and found that transgene expression was markedly stimulated independently of transgene, promoter and type of vector [46]. Thirdly, lentiviral vectors were further shaped by the finding that a short motif in the vector consisting of the central polypurine tract and central termination sequence, involved in initiation and termination, respectively, of DNA synthesis during reverse transcription, was a key determinant for nuclear import of the vector [47–49]. Although the exact function of this element, often referred to as cPPT/FLAP remains somewhat controversial [50–55] it is maintained in standard lentiviral vectors due to the beneficial effect on transduction efficacy. Fourthly, insulators and chromatin opening elements have been inserted into lentiviral vectors to protect the transgene from chromosomal position effects and transcriptional silencing through the spread of heterochromatin. The 5′-HS4 β-globin (cHS4) insulator, which may both protect the transgene cassette against silencing and block the interactions between a transgene promoter and neighboring promoter and enhancers [56], has been found to support increased and stable levels of transgene expression from integrated lentiviral vectors [57]. Another heterologous element, the ubiquitous chromatin-opening element (UCOE) derived from a human CpG island containing bidirectional promoters in the HNRPA2B1-CBX3 locus has also attracted attention as an element protecting against transcriptional silencing [58]. On several occasions, UCOE has been observed to support stable transgene expression in cells transduced with lentiviral vectors carrying an UCOE-supported transgene expression cassette [59–61].

### Tailored lentiviral vectors in hematopoietic stem cell gene therapy

**Fig. 5:**
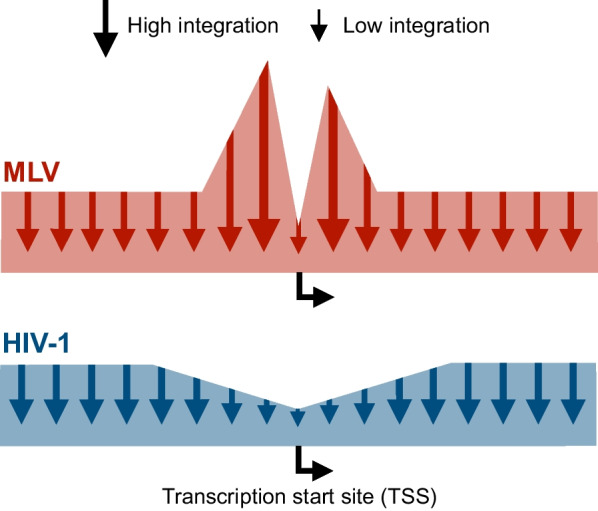
Schematic representation of MLV and HIV-1 integration profiles. The integration profile of retroviral vectors derived from murine leukemia virus (MLV) exhibits a strong bias for regions flanking transcription start sites (TSS), whereas lentiviral vectors derived from HIV-1 does not exhibit the same preference for integration near transcription start sites, but instead have a preference towards actively transcribed regions. Schematic representation based on data presented by Cattoglio et al. [74]

**Fig. 6:**
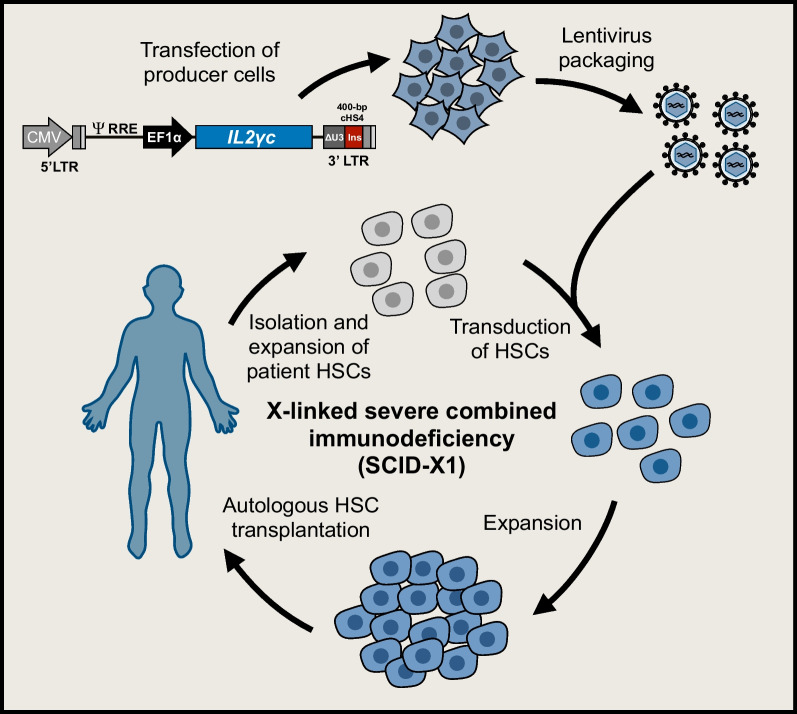
Schematic overview of lentiviral gene therapies used for the treatment of SCID-X1. The strategy employed to treat X-linked severe combined immunodeficiency (SCID-X1) is based on the delivery of a normal copy of the *IL2γc* gene into the genome of a patient’s own hematopoietic stem cells (HSCs). HSCs are isolated from the patient and expanded prior to transduction using a lentiviral vector carrying a normal cDNA copy of the *IL2γc* gene expressed from an EF1α promoter. In addition to using the SIN-configuration, the lentiviral vector contains a 400-bp chicken β-globin insulator element, which aids in the safety of the vector by contributing enhancer-blocking activity. Integration of the ‘healthy’ *IL2γc* gene into the patient's HSCs restores *IL2γc* expression in HSCs, which upon autologous transplantation are able to reconstitute functional immunity

Soon after the early reports on lentiviral gene delivery, it became evident that early-generation VSV-G-pseudotyped HIV-1-derived vectors facilitate efficient gene transfer to CD34 + hematopoietic stem cells (HSCs) derived from umbilical cord blood and that transduced cells are capable of engrafting in NOD/SCID mice [62]. This elicited an immediate interest of using lentiviral vectors for clinical gene transfer to CD34 + cells, but concerns related to the impact of vector integration and insertional mutagenesis were reinforced by the emerging leukemia cases in patients treated with gammaretroviral vectors. This focused the attention on the integration profile of lentiviral vectors. Initial work by the Bushman group brought worrying news by documenting the tendency of HIV-1 to integrate primarily in actively transcribed genes [63]. Based on lentiviral transduction of peripheral blood mononuclear cells and lung fibroblast, up to 80% of integration sites were found to map to active genes [64]. However, these studies also revealed a crucial difference between MLV- and HIV-1-derived vectors, namely that HIV-1 did not, like MLV, integrate into transcriptional start sites, but had a tendency to find other positions along the full length of the gene to integrate [64]. This difference reflects that MLV and HIV-1 interact with different cellular proteins en route to the integration site. Thus, a series of papers have described a key role of LEDGF/p75, which interacts with HIV-1 integrase and helps guide the integration process and the insertion profile in genes [65–68]. Indeed, several studies have modulated the insertion profile of HIV-1-based lentiviral vectors by altering the chromatin binding domain of LEDGF/p75 to redirect vector integration to other sites [69–73]. Focusing on the inherent properties of unmodified vectors, a key study mapped > 32,000 MLV vector integration sites and > 28,000 HIV-1 vector integration sites in human CD34 + hematopoietic progenitor cells, confirming that MLV insertion sites clustered in gene-regulatory regions whereas HIV-1 vector insertions mapped primarily within genes (75.7%). However, whereas MLV integration sites clustered around transcription start sites, HIV-1 insertions were significantly reduced in the same region and equally distributed to other regions of the targeted genes (Fig. 5) [74]. A sigh of relief went through the gene therapy community. Such findings indeed supported the notion that lentiviral vectors are safer than MLV vectors cursed with detrimental side effects, and this sparked a new series of study lines aiming at clinical translation of gene therapy in CD34 + hematopoietic stem cells using optimized vector configurations and a standard clinical approach (Fig. 6).

**Fig. 7:**
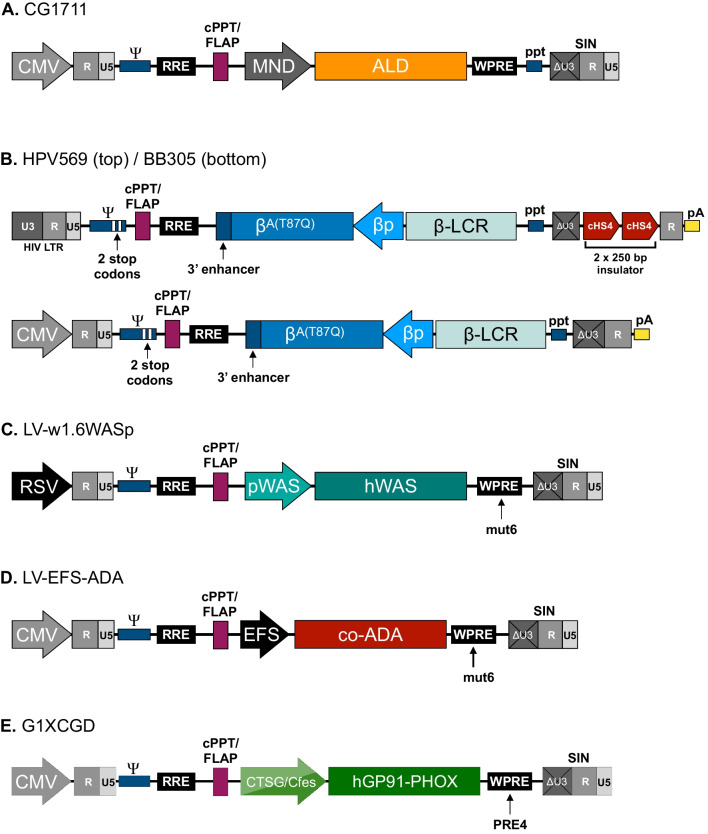
Examples of lentiviral vectors used in clinical studies. **A** The CG1711 vector used by Cartier et al. for the treatment of ALD. **B** The HPV569 and BB305 LentiGlobin vectors used for the treatment of β-thallassemia. **C** The LV-w1.6WASp used to treat WAS. **D** The LV-EFS-ADA vector used for the treatment of ADA-SCID. **E** The G1XCDG vector used for the treatment of X-CGD. See text for details. All vectors are shown schematically, and elements such as genes and promoters are not shown to scale

Lentivirus-based hematopoietic stem cell gene therapy has been used to treat X-linked adrenoleukodystrophy (X-ALD) [75], the lysosomal storage disease metachromatic leukodystrophy (MLD) [76, 77], β-hemoglobinopathies like β-thallassemia [78–80] and sickle-cell disease [81], and not least primary immunodeficiencies including ADA-SCID [82], SCID-X1 [83], Wiskott-Aldrich Syndrome (WAS) [84–86], and X-linked chronic granulomatous disease (X-CGD) [87]. It is beyond the scope of this review to cover details leading to clinical translation of gene therapies, and the reader is referred to several excellent reviews by authors who are or have been key players in the clinical trials [26, 82, 88–91]. However, common for the implementation of lentiviral vectors in patients was years of thorough analyses of tailored vector configurations featuring ex vivo transgene expression studies, in vitro transformation assays, integration profiling, and preclinical in vivo studies in relevant mouse models. Notably, despite obvious clinical differences between the diseases, VSV-G-pseudotyped third-generation SIN vectors were utilized to transduce the same target: patient-derived CD34 + hematopoietic stem cells. In Fig. 7, we provide examples of vector designs that made it to the clinic.

X-ALD results from mutations in the *ABCD1* gene encoding the ALD protein, an adenosine triphosphate-binding cassette transporter. For treatment of X-ALD, Cartier and co-workers used a vector design which was very similar to the original SIN design using the pCCL configuration (Fig. 7A). ALD protein was produced from the wildtype *ABCD1* cDNA driven by a modified myeloproliferative sarcoma virus promoter (referred to as MND), and expression of the transferred *ABCD1* gene was detectable in patients > 2 years after transfusion, leading to amelioration of key neurological symptoms. The β-hemoglobinopathies are commonly caused by defects in the production of β-globin, and so efforts at treating β-hemoglobinopathies using lentiviral gene therapy have revolved around transfer of a healthy β-globin gene. For production of β-globin for treating β-thalassemia, so-called LentiGlobin vectors have been used (Fig. 7B). An early version of this design, HPV569, was a SIN vector containing two copies of the 250-bp core of the cHS4 insulator inserted in the U3 region of the 3’LTR to combat potential insertional mutagenesis by insulating nearby protooncogenes from activation [92]. Expression of this vector during packaging was driven by HIV-1’s own promoter, and the transgene cassette, placed in the reverse orientation relative the vector itself, encoded a mutated adult β-globin variant (β^A(T87Q)^) driven by the human β-globin promoter flanked by the human β-globin Locus Control Region. The use of a mutated adult β-globin variant allowed expression of the transferred gene to be distinguished from transfused wild-type β-globin in the patients [93]. For further safety, this vector featured two stop codons in the Ψ sequence and a heterogenous poly A sequence. In a modified version of this vector, called BB305 used for subsequent clinical trials, the two insulator core elements were removed, and a pCCL-style hybrid CMV-LTR promoter was used for production of vector RNAs [80] (Fig. 7B). LentiGlobin BB305 was also used for treatment of a patient suffering from Sickle Cell Disease resulting in high levels of antisickling β-globin and correction of key hallmarks of the disease > 1 year after treatment [81].

WAS is a primary immuno-deficiency caused by mutations in the *WAS* gene. In protocols aiming at treating WAS, a vector typically referred to as LV-w1.6WASp has been used across several trials (Fig. 7C). This vector is based on a format developed by Dupre et al., who tested transgene expression controlled by different variants of the endogenous promoter driving expression of the *WAS* gene [94]. Further studies confirmed that the 1.6 kb fragment upstream from the transcriptional start site of the *WAS* gene results in high levels of transgene expression in hematopoietic cells, comparable to levels seen with classic heterologous promoters, demonstrating the possibility of utilizing the native regulatory sequence to obtain cell-targeted expression of the *WAS* cDNA [95]. A final version of the vector contains a mutated variant of the WPRE signal, WPREmut6, which supports long-term transgene expression but carries a mutation disturbing the open reading frame (ORF) present in the wildtype WPRE sequence [96]. The ORF in question encodes a truncated Woodchuck hepatitis virus X protein (WHV-X), which is potentially oncogenic [97] and thus safety of the LV-w1.6WASp vectors was increased by abrogating WHV-X protein synthesis. Patients treated with this vector have shown stable engraftment of genetically corrected hematopoietic stem cells leading to sustained clinical benefit and improved immune functions as evident from improvements in eczema and the frequency and severity of infection [84, 85]. Most recently, a patient treated with this type of vector was able to discontinue immunosuppression and support with immunoglobulins [86]. Based on the experience with this vector, a pCCL-type vector for clinical use for treatment of ADA-SCID has been produced by exchanging the W1.6-WAS cassette with a codon-optimized version of the *ADA* cDNA under transcriptional control of the short form of constitutively acting elongation factor 1α promoter (EFS) (Fig. 7D) [98]. The efficacy of this vector was recently demonstrated in a clinical trial for ADA-SCID that showed high overall survival with sustained ADA expression and functional immune reconstitution [26, 99]. X-CGD results from variants in the *CYBB* gene encoding gp91^phox^, a catalytic subunit of the phagocyte NADPH-oxidase. For treatment of patients, a pCCL-type lentiviral vector containing a codon-optimized CYBB *cDNA* version was expressed from a chimeric promoter allowing high levels of expression in myeloid cells [87] (Fig. 7E). This promoter, a fusion of 5’-flanking regions of the genes encoding cathepsin G and c-Fes, is highly active in granulocytes and was found in this vector context to effectively restore NAPDH-oxidase activity [100].

### Lentiviral vector integration site distribution: experience from the clinic

Based on the above-mentioned clinical studies representing ten years of experience with utilizing lentiviral vectors to deliver therapeutic transgenes to hematopoietic stem cells, the safety of lentiviral integration of genes into stem cells can be evaluated. Due to technical improvements of next-generation sequencing methodologies, the number of vector integration sites identified in individual patients has steadily increased from the first clinical trials, and the combined number of mapped vector integration sites (for each study counting only sites that are unique for that particular study) now totals more than 1.5 million integration sites. Most recently, Kohn and co-workers identified 724,685 unique lentiviral integration sites in nine X-CGD patients [87]. Analyses of vector integration site distribution allows monitoring of the extent of polyclonal hematopoiesis, and integration sites can be used to track clonal behavior and potential clonal expansion. Notably, all studies confirmed the tendency of lentiviral vectors to insert within genes (often between 70 and 80% of insertions are mapped in genes) and to cluster in gene-rich regions. Encouragingly however, in all studies, except one, polyclonal integration profiles without detection of dominant clones were documented indicative of an absence of clonal outgrowth and a reduced risk of engrafting lentivirally transduced stem cells. In one trial, a benign clone carrying a vector integration in the *HMGA2* gene was created and found to support therapeutic benefit by disrupting regulation of the transgenic β-globin gene leading to increased production [78]. This dominance has not been associated with any serious adverse events and was found to be progressively replaced by other clones by year 12 after treatment [80]. Together, reports from clinical trials demonstrate polyclonal repopulation of hematopoiesis without signs of genotoxicity and argue that integration profiles of third-generation lentiviral vectors are indeed safer than the profiles of gammaretroviral vectors. These findings support continued development of lentiviral gene transfer for stem cell therapies.

### Toward in vivo applicability of lentiviral vectors

For most diseases, it is not an option to handle cells ex vivo and transfer therapeutic genes in culture flasks. Wisely, Friedmann and Roblin urged to caution in treating human disease by administering genes directly to patients when they first framed the concepts of gene therapy almost 50 years ago [101]. Indeed, over the years the gene therapy community has experienced some of the scientific challenges that Friedmann and Roblin foresaw, but numerous breakthroughs, primarily founded on adeno-associated virus (AAV)-based gene delivery, and not least the discovery of novel genome editing technologies, have attracted further attention and massive investments to develop in vivo gene therapies. Early on, the series of papers that eventually led to third-generation lentiviral vectors consistently showed robust gene delivery to terminally differentiated neurons in rats injected with VSV-G-pseudotyped lentiviral vectors into the striatum or hippocampus [38, 39, 44, 102, 103]. Later studies showed effective lentiviral gene marking in adult neural stem cells in the subventricular zone of the adult mouse brain [104, 105]. Also, in rat eyes injected subretinally with GFP-encoding lentiviral vectors, effective transduction and GFP expression was evident in both retinal pigment epithelium cells and photoreceptor cells using CMV and rhodopsin promoters, respectively [106]. This strategy was recently utilized to deliver genome editing tool kits to mouse retinal pigment epithelium cells to achieve knockout of the *vegfa* gene as a feasible treatment of age-related macular degeneration [107].

Whereas delivery of transgenes to cells in vivo has mostly been achieved using lentiviral vectors pseudotyped with VSV-G due its broad tissue-specificity and high transduction capacity, an intriguing aspect of in vivo lentiviral vector delivery is the ability to replace VSV-G with alternative surface proteins, thereby altering vector tropism. Girard-Gagnepain and coworkers demonstrated incorporation of the baboon endogenous retrovirus glycoprotein on the surface of virions, resulting in increased vector transduction efficacy in human CD34 + HSCs, B-cells, and T-cells compared to VSV-G-pseudotyped lentiviral vectors [108]. As an additional example, Kobinger and coworkers showed efficient transduction of airway epithelial cells in vivo using lentiviral vectors pseudotyped with Filovirus envelope protein [109].

For liver-directed gene therapy, AAV-based vectors have shown immense clinical potential leading for example to sustained therapeutic expression of highly active factor IX in AAV-treated hemophilia B patients [110]. However, lentiviral vectors could potentially offer certain advantages, for example by allowing transgene integration in growing livers of pediatric patients. Also, whereas AAV-directed gene therapy may be challenged by preexisting neutralizing anti-AAV antibodies and cellular immunity against AAV, such preexisting immunity against pseudotyped HIV-derived particles is less frequent in patients. Failure of early attempts of achieving stable factor IX in lentivirally transduced mouse liver, led Brown and coworkers to develop lentiviral vectors carrying target sites for hematopoietic-specific microRNAs [111]. The rationale of this elegant approach was to restrict vector expression posttranscriptionally in transduced hematopoietic cells including antigen-presenting cells of the immune system, thus minimizing the risk of eliciting an immune response against transgene-encoded factor IX [111]. As a result, administration of lentiviral vectors harboring an array of four miR-142-3p target sites led to phenotypic correction in hemophilia B mice [112]. Notably, injection of such lentiviral vectors in mouse models prone to develop liver cancer by insertional mutagenesis did not lead to cancer formation, arguing that genomic integration of vectors was safe and did not cause genotoxicity [113]. Similar approaches were utilized to treat rodent models of hyperbilirubinemia [114] and hemophilia A [115]. Using the miR-142-3p-regulated vector configuration, stable reconstitution of Factor IX (up to 1% of normal) was achieved in three hemophilia dogs leading to a reduction of spontaneous bleeding events in all three animals treated with in vivo lentiviral gene therapy [113]. However, this relatively modest outcome was hypothesized to reflect rapid clearance of systemically administered lentiviral particles [116], which led to studies touching on lentiviral production methods and in particular the engineering of the producer cell-derived plasma membrane surrounding the virus particle. Hence, Milani and coworkers figured that the density of CD47 molecules, an inhibitor of phagocytosis in humans, has importance for the ability of virus particles to evade uptake by phagocytosis by liver and spleen macrophages as well as antigen-presenting cells. They engineered cell lines with overexpression of CD47 and found that CD47-loaded lentiviral vectors produced in these producer cells showed lower susceptibility to phagocytosis resulting in increased levels of transgene expression in nonhuman primates [116]. These findings illustrated both safety and efficacy in a close-to-human model and pointed to the choice of producer cell and in particular engineering of the plasma membrane as key targets for optimizing lentiviral vector performance in vivo [117].

### Use of lentiviral vectors for production of CAR-T cells

The clinical potential of lentiviral vectors has been particularly apparent in the generation of chimeric antigen receptors (CAR) T-cells, which have shown an impressive potential for treatment of hematological malignancies [118–121]. CARs are synthetic receptors consisting of an extracellular immunoglobin domain to impart antigen recognition as well as both an intracellular T-cell activating domain (typically CD3ζ) and additional co-stimulatory signaling domains such as CD28 or 4-1BB [122]. Using gene transfer techniques, these engineered CARs can be delivered to immune effector cells, most notably autologous T-cells, redirecting the specificities of the cells, which upon infusion and engraftment are able to exhibit anti-tumor effects [123]. While modern CAR T-cells have been generated using both gammaretroviral and lentiviral vectors as well as non-viral vectors, lentiviral vectors have become the vectors of choice for generation of CAR T-cell therapies due to the favorable integration profile and high transduction rates [124]. It is not our intention here to give a detailed overview of the use of lentiviral vectors in CAR T cell therapies, but simply to give flavor of the importance of these vectors for CAR-T cell engineering. The CD19-targeted CAR-T cell therapy Tisagenlecleucel (Kymriah™, formerly known as CTL019), which was FDA approved in 2017 and is now used for the treatment of B-cell precursor acute lymphoblastic leukemia (ALL) and relapsed or refractory (r/r) large B-cell lymphoma, is a prominent example of a CAR-T therapy produced using lentiviral gene transfer [119, 125–130]. Generation of these anti-CD19 CAR-T cells was accomplished using third-generation pRRL-type SIN lentiviral vectors, essentially a standard setup, designed to deliver the anti-CD19 CAR into stimulated CD4 and CD8 T cells [129]. While developing anti-CD19 CAR T-cells, Milone and coworkers further demonstrated that expression of the anti-CD19 CAR could be increased and optimally maintained in both CD4 and CD8 T cells by replacing the internal CMV promoter in the pRRL-type vector with the elongation factor-1α (EF-1α) promoter, which is crucial for the long term antitumor effects of CAR T-cells in vivo [129].

Another area where CAR-T cells has shown clinical efficacy is in the treatment of neuroblastoma using anti-GD2 CAR T-cells. However, compared to the response in B-cell malignancies, clinical translation of CAR T-cell therapy in solid tumors such as neuroblastoma has not been quite as successful reflecting various challenges related to solid tumors, including the lack of highly expressed tumor-specific antigens as well as immunosuppressive tumor microenvironments, leading to exhaustion and dysfunction of infused CAR-T cells [131–133]. However, one of the strategies developed to remedy the ineffectiveness of CAR T-cell therapy in the treatment of solid tumors was to equip the CAR T-cells with an additional inducible cytokine response, typically mediated through the nuclear factor of activated T-cells (NFAT) transcription factors. The development of such so-called TRUCKs (T cells redirected for universal cytokine-mediated killing) can be generated by delivery of two separate vectors; one encompassing the CAR and one encompassing the NFAT-inducible cytokine (e.g. IL-12). However, recently Zimmermann and coworkers utilized the large cargo capacity of lentiviral vectors to generate anti-GD2 TRUCKs using a single third-generation SIN lentiviral vector to transfer both a constitutively expressed anti-GD2 CAR as well as a NFAT-inducible IL-12 [134].

Given that the design of state-of-the-art lentiviral vectors has not changed dramatically since the development of the third-generation lentiviral vectors, virtually all efforts including lentiviral vectors for production of CAR T-cells have been based on the third-generation packaging system with the SIN configuration. However, early version second-generation lentiviral vectors were indeed used in studies that went on to clinical trials [126]. Given the enormous attention on CAR T-cell therapies and the rapid growth of CAR-T-based immunotherapies, standard third-generation lentiviral vectors will continue to play a key role in development of cancer therapies.

### CRISPR delivery using lentiviral vectors

Genome editing based on a rapidly growing family of CRISPR technologies has revolutionized molecular biology and is quickly being transformed into promising tools for site-directed gene modification and gene therapy [135]. CRISPR gene editing based on the standard CRISPR/Cas9 system is based on the co-delivery of the Cas9 endonuclease and a single guide RNA, the latter which guides Cas9 to a predetermined region in the genome through base pairing between the sgRNA and one of the two DNA strands in the target locus [136]. In addition, an exogenous donor is required, if gene modification involves repair of the cleavage site through homologous recombination. As CRISPR delivery has become a key focus, the gene therapy community has, not surprisingly, found inspiration in vector technologies developed to meet the requirements in conventional gene therapies. However, at least for therapeutic purposes, the goal has changed; with CRISPR, the aim is to obtain efficient gene transfer allowing Cas9/sgRNA complexes to build up quickly in targeted cells, but expression should be only transient, as prolonged production of Cas9/sgRNA may be toxic due to immune responses or increased levels of off-target DNA cleavage.

**Fig. 8:**
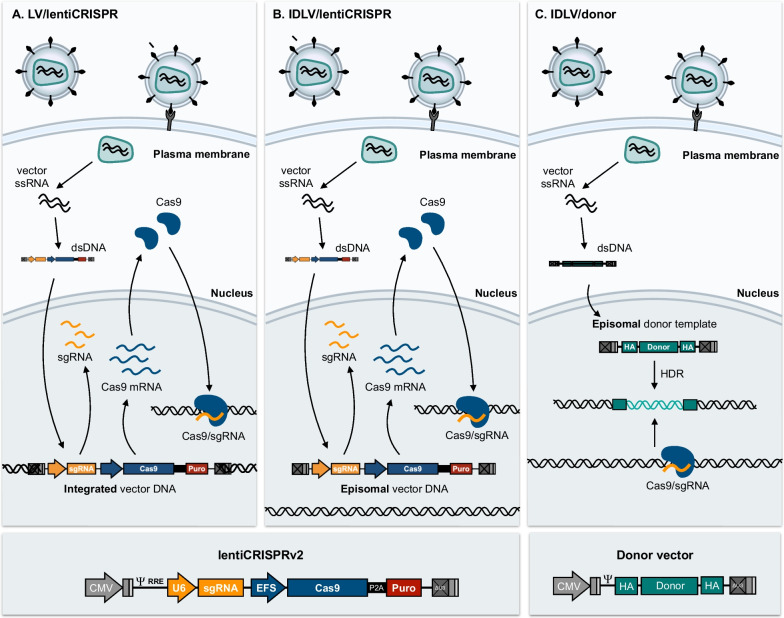
Schematic overview of CRISPR/Cas9 delivery using lentiviral vectors. **A** Cas9 and sgRNA are delivered within a single lentiviral transfer vector (lentiCRISPR-v2). Integration of the lentiCRISPR-v2 vector in the genome of target cells results in persisting expression of both Cas9 and sgRNA, which can then act on the genome of target cells. **B** Using integrase-defective lentiviral vectors (IDLVs), the lentiCRISPR-v2 vector remains episomal upon transduction, which results in only transient expression of both Cas9 and sgRNA, thus minimizing potential unwanted off-target effects. **C** In addition to delivering Cas9 and sgRNA, IDLVs can also be utilized to deliver a repair template (donor) for homology-directed repair (HDR)

Gene transfer based on vectors derived from adeno-associated virus (AAV) is efficient, safe, and well-studied, and these vectors are being investigated for co-delivery of Cas9 and sgRNA expression cassettes [137–139] as well as for delivery of donor repair templates [140–142]. In growing cells, AAV-based CRISPR delivery is likely to lead to transient production of Cas9/sgRNA complexes due to the loss of episomal DNA intermediates over time, whereas expression in non-dividing cells may be long-term and potentially toxic, if expression is not regulated and restricted. Lentiviral vectors have become important carriers of CRISPR tools, but genomic integration of Cas9 and sgRNA expression cassettes leading to permanent expression is not likely to support safe therapeutic use, unless expression is tightly regulated. Therefore, based on the lentiCRISPRv2 vector published by the Zhang lab [143], lentiviral delivery has primarily evolved as a crucial tool in molecular genetics research. LentiCRISPRv2 is a classical pCCL-type third-generation lentiviral vector carrying two expression cassettes, (i) a sgRNA cassette driven by the U6 snRNA promoter, which is a Type III RNA polymerase III promoter commonly used for driving expression of small RNAs and (ii) a cassette with an elongation factor-1α factor short promoter driving expression of *Streptococcus pyogenes* Cas9 (SpCas9) fused to a marker gene through a 2A self-cleaving peptide (Fig. 8). An earlier v1 version of this vector resulted in relatively low titers [144], but a series of smaller adjustments, including repositioning of the sgRNA cassette, led to LentiCRISPRv2, which produces vectors with higher functional titers [143]. For specific research purposes, it may be helpful to produce cell lines with stable expression of SpCas9 and utilize lentiviral vectors to deliver the sgRNA cassette only along with a selection marker gene. The transfer efficiency of this vector is higher than vectors carrying the SpCas9 gene, which may be crucial for some applications [143]. Genome editing based on transduction of standard lentiviral vectors is based on complex formation between SpCas9 and sgRNA produced from an integrated copy of the vector (Fig. 8A). In a cell population selected for the presence of an integrated vector, targeted indels will appear over time, as SpCas9/sgRNA complexes build up and eventually cleaves DNA in the desired location. Depending on the cell type, indels may start to appear after 24 h and accumulate over the next days, typically resulting in complete knockout, if the target site is located in the coding region of a gene [145].

**Fig. 9:**
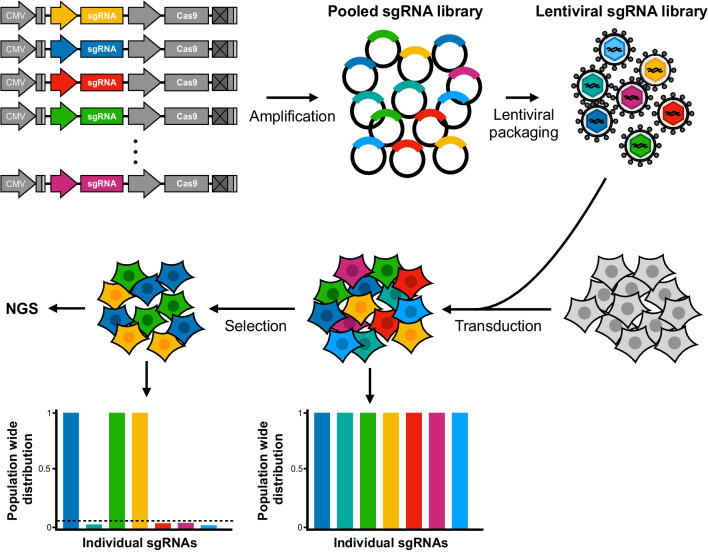
CRISPR-based lentiviral knockout (KO) libraries. A library of pooled sgRNAs are cloned into a suitable lentiviral vector (here exemplified by a lentiCRISPR-v2 vector), resulting in a plasmid library, which is then used for production of a pooled lentiviral sgRNA library. Transduction of target cells is made at a low MOI ensuring that each cell receives a single unique sgRNA. Transcription of sgRNA and Cas9 results in KO of the target gene in each transduced cell, which results in a large population of cells collectively carrying knockout mutations in all library-targeted genes. Selection (e.g. a chemotherapy drug) is then applied to the pool of cells, which makes it possible to detect genes that affect the drug response. Genes involved in the given drug response is identified by next-generation sequencing (NGS) of sgRNA-containing cassettes and downstream bioinformatic analysis

Genomic insertion of SpCas9- and sgRNA-encoding lentiviral vectors is an essential feature of novel lentiviral CRISPR-based strategies for interrogating gene function. By combining the properties of lentiviral vectors to integrate with CRISPR-directed gene knockout, it is possible to create genome-wide lentiviral CRISPR libraries consisting of thousands of different lentiviral vectors each encoding a unique sgRNA targeting a single gene [144]. Lentiviral transfer of a sgRNA library to a population of cells with stable expression of SpCas9 leads to the emergence of different knockout mutations in each cell determined by the sgRNA expressed in that particular cell (Fig. 9). This creates a heterogenous population consisting of cells, each carrying a unique genotype as well as a lentiviral ‘footprint’, or barcode, with a sgRNA sequence revealing the identity of the targeted gene. Depending on the selection modality, e.g. resistance to cancer drugs, applied to the heterogenous cell population, candidate genes affecting a phenotype of interest can now be identified by sequencing of the pool of sgRNA cassettes present in the selected population. Such genome-wide screens have identified genes driving tumor growth and cell proliferation in various cancers [144, 146]. We and others have identified genes affecting drug responses on cancerous B-cells in diffuse large B-cell lymphoma [147–149] and provided protocols for carrying out genome-wide screens utilizing integrating lentiviral vectors [150–153].

For specific purposes, it may be attractive to deliver the CRISPR components without integrating the SpCas9 and sgRNA genes in the genome (Fig. 8B). Early studies of a series of HIV-1 integrase mutants showed reduced integration but unaffected production of episomal DNA intermediates in cells infected with viruses carrying amino acid substitutions, e.g. D64V, in the catalytic domain of the integrase [154]. Thus, integration of viruses carrying the D64V integrase variant was reduced to 1/10.000 of the activity in wildtype viruses. Third-generation integrase-defective lentiviral vectors (IDLVs) produced with packaging constructs carrying the D64V variation were originally shown to facilitate effective gene transfer and stable expression in postmitotic tissues in vivo [155] and have been adapted for delivery of alternative integration platforms based on the transient expression of recombinases [156] and DNA transposases [157–159]. In case of CRISPR delivery, lentiCRISPRv2 can be delivered in an IDLV context, allowing only transient SpCas9/sgRNA expression, at least in dividing cells (Fig. 8B). Lombardo and coworkers adapted the IDLV configuration for delivery of zinc-finger nucleases to hematopoietic stem cells [160], and Ortinski et al. adapted the LentiCRISPRv2 vector for IDLV-directed delivery of SpCas9 and sgRNA to the ventral striatum in rats, demonstrating long-term effects of targeted gene knockout after IDLV delivery in non-dividing brain cells [161]. IDLV vectors allow introduction of indels without integrating the vector in the genome, but may in dividing cells in particular come with the risk of reduced efficiency relative to integration-proficient systems. Notably however, for CRISPR-based genome editing the IDLV platform may be particularly suitable for delivery of episomal donor repair templates, which may upon reverse transcription in transduced cells be available for repair by homologous recombination, allowing specific edits to be introduced in the genome (Fig. 8C) [160, 162].

### Lentiviral protein delivery: a new path for lentiviral transport

The therapeutic focus of gene therapy has always been on delivery of genetic information to cells and tissues. However, with the discovery of genome editing technologies based on the administration of genome editing tool kits, the ultimate goal may not necessarily be long-term expression of transgenes but rather potent, but short-term activity of proteins at work in the genome. Not unexpectedly, attempts of translating CRISPR editing methodologies into therapies have so far, as noted above, found inspiration in the potent gene delivery technologies, which have been developed and optimized over the last 40 years. However, to reduce off-target cleavage events and avoid toxic effects, these techniques may require strict regulation of gene expression, allowing production of Cas9 and sgRNA to be shut-down after successful genetic intervention, and may not eventually be the best agents for therapeutic CRISPR administration.

**Fig. 10:**
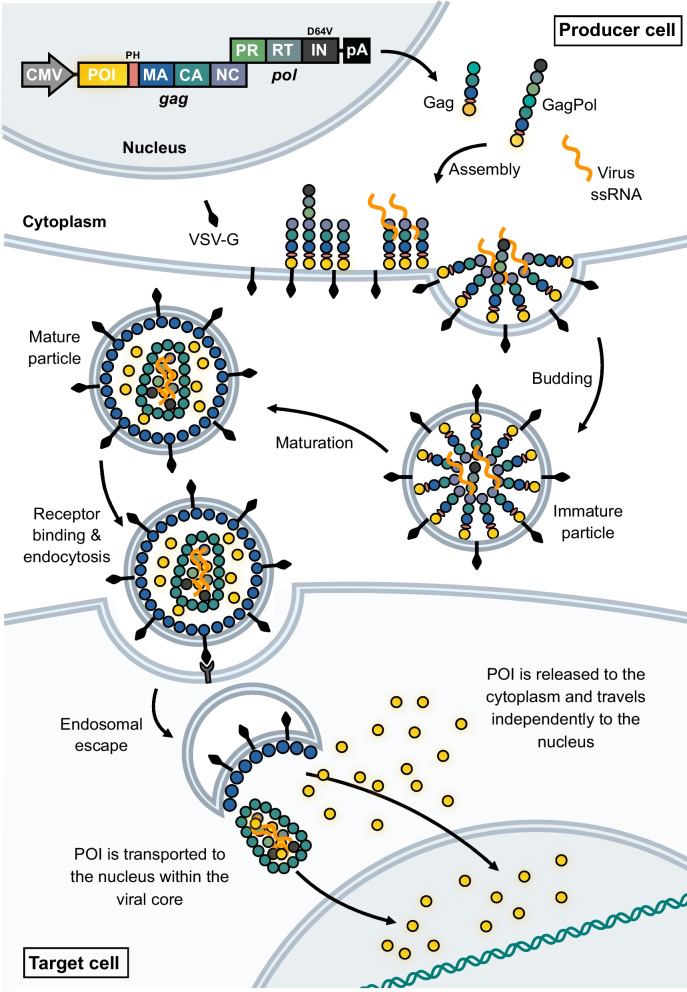
Schematic representation of lentiviral protein transduction. Lentiviral vectors can be manipulated to package a protein of interest (POI). This can be done either by fusing the POI to the C-terminal integrase (IN) protein of the GagPol polypeptide, or as shown in this illustration, by fusing it to the N-terminal matrix (MA) protein separated by a Pleckstrin homology domain (PH) to aid the anchorage to the plasma membrane. The integrase harbors the D64V mutation rendering the viral particles integrase-defective. By including a protease recognition site between the POI and MA protein, the POI is released in mature viral particles and delivered to the nucleus of the target cell by mechanisms currently unknown. Two possible mechanisms have been suggested: Transport of the POI within the viral core or diffusion through the cytoplasm. An optional vector RNA genome can be included in the viral particles, which in interest of CRISPR-Cas9 genome editing, could be a donor template for HDR. In this case, the lentiviral particles would carry all necessary components for CRISPR-based HDR within a single lentiviral particle

Whereas nucleofection of recombinant Cas9 protein complexed with chemically modified sgRNAs has become a preferred strategy for ex vivo genome editing of stem cells [140], there is still room for improvement of in vivo delivery technologies that can be targeted to cell types and offer potent, but short-term, exposure of target cells to the CRISPR tools. Yet again, viruses are demonstrating how delivery, now of proteins, can be accomplished, lending inspiration to the development of protein delivery tools. Retro- and lentiviruses are characterized by their ability to convert RNA to DNA by reverse transcription and to insert double-stranded DNA into host cell genomes. These processes are governed by reverse transcriptase and integrase, respectively, proteins which the virus itself brings along with genetic information into host cells. Such properties of the virus can be harnessed to allow virus particles to incorporate and transfer foreign proteins, including genome-modifying enzymes, into the nucleus of cells exposed to protein-engineered virus particles. By fusing foreign proteins of interest to the N-terminus of Gag and GagPol protein, we have shown that such proteins are incorporated into virus particles, are released from the viral polypeptides during virion maturation in a protease-dependent fashion, and released inside cells in processes involving endosomal escape (see Fig. 10 for schematic representation) [163]. In previous work, we demonstrated transfer and functionality of piggyBac DNA transposase, zinc-finger nucleases, and TAL-effector nucleases packaged and transported to cells in lentivirus-derived particles [162–165]. Moreover, lentiviral delivery of Cas9 protein induces formation of targeted indels in cells expressing the sgRNA [166], suggesting that lentiviral delivery can be further optimized to facilitate transfer of ribonucleoprotein complexes consisting of Cas9 and sgRNA. In earlier work, we showed gene correction in cells treated with ‘all-in-one’ lentiviral particles carrying both locus-targeted endonucleases and vector RNA containing the repair template for homologous recombination upon reverse transcription [162]. As this approach for delivery of genome editing tool kits is still in its infancy, evidence of in vivo applicability is still lacking, but the potential for performing cell-targeted genome editing using different pseudotypes and delivering more sophisticated editing technologies, like RNA-guided base- and prime-editors [167, 168], encourages further investigations of such approaches.

## Conclusion

Somewhere back in time, viruses evolved to transport their own genetic information and carry the proteins required to process the genetic information in host cells. We now know that viruses interact with cellular factors not only to fight cellular antiviral responses, but also to traverse the cytoplasm and gain access to the nucleus and potentially to attractive landing sites in the genome. Such early steps of viral evolution have shaped viruses and provided properties that may either support or hamper their use as vector technologies for safe clinical gene therapy. HIV-derived lentiviral vectors were endowed with a desirable integration profile facilitating the landing of transgene cassettes in regions of transcriptional activity without severely impacting endogenous gene expression. Emerging evidence from clinical trials bear witness of a safe and potent vector technology with obvious clinical benefit in both gene and cell therapies. At the same time, new ways of exploiting lentiviral delivery for genome-wide screening and genome editing are sprouting. Lentiviral vectors are in their prime—and remain state-of-the-art after 25 years.

## Data Availability

Not applicable.

## Abbreviations

AAV: Adeno-associated virus
ADA: Adenosine deaminase deficiency
AIDS: Acquired immunodeficiency syndrome
BET: Bromodomain and extra-terminal
CAR: Chimeric antigen receptor
Cas9: CRISPR-associated protein 9
CMV: Cytomegalovirus
cPPT: Central polypurine tract
CRISPR: Clustered regularly interspaced short palindromic repeats
EF-1α: Elongation factor-1α
EFS: Short elongation factor 1α promoter
HIV-1: Human immunodeficiency virus type 1
HSC: Hematopoietic stem cell
IDLV: Integrase-defective lentiviral vector
LTR: Long terminal repeat
MLD: Metachromatic leukodystrophy
MLV: Murine leukemia virus
Mo-MLV: Moloney-MLV
ORF: Open reading frame
R-MLV: Rauscher-MLV
RSV: Rous sarcoma virus
RT: Reverse transcriptase
SCID-X1: X-linked SCID
SCID: Severe combined immunodeficiency
sgRNA: Single guide RNA
SIN: Self-inactivating
UCOE: Ubiquitous chromatin-opening element
VSV-G: Vesicular stomatitis virus glycoprotein
WAS: Wiskott-Aldrich Syndrome
WHV-X: Woodchuck hepatitis virus X
WPRE: Woodchuck hepatitis virus posttranscriptional regulatory element
X-ALD: X-linked adrenoleukodystrophy
X-CGD: X-linked chronic granulomatous disease
